# Do Self- and Proxy Reports of Cognitive Problems Reflect Intellectual Functioning in Children and Adolescents with Congenital Heart Defects?

**DOI:** 10.3389/fped.2016.00127

**Published:** 2016-11-28

**Authors:** Sandra Buratti, Carmen Ryberg, Malin Broberg, Jan Sunnegårdh

**Affiliations:** ^1^Department of Pediatric Cardiology, The Queen Silvia Children’s Hospital, Sahlgrenska University Hospital, Gothenburg, Sweden; ^2^Department of Psychology, University of Gothenburg, Gothenburg, Sweden

**Keywords:** intellectual functioning, neurodevelopment, congenital heart defects, cardiac treatment by surgery or by catheter interventions, quality of life, self-report, proxy reports

## Abstract

**Aim:**

Children with congenital heart defects (CHD) who suffer from cognitive impairments and school difficulties need to be identified as early as possible in order to set appropriate interventions in place that may enhance the school situation and quality of life for these children. Identifying children and adolescents at risk for cognitive difficulties requires specific screening tools. This study assessed such a tool – Pediatric Quality of Life Inventory Cardiac Module subscale: Cognitive Problems – to investigate whether proxy reported and self-reported cognitive problems were associated with measured intellectual functioning in children and adolescents with CHD treated with surgery or by catheter interventions.

**Method:**

The sample consisted of 184 children/adolescents aged 3, 5, 9, and 15 years. The severity of the CHD diagnoses was categorized into three groups (mild, moderate, or severe) for all age groups. For all the age groups, we collected proxy ratings of cognitive problems, and for the 5-, 9-, and 15-year-olds, we also collected self-reported cognitive problems. Intellectual functioning was measured with the Wechsler intelligence scales. The control variables were socioeconomic status and severity of diagnosis.

**Results:**

A strong association was found between the parent’s ratings of cognitive problems and the children’s and adolescents’ results on the Wechsler scales. This association was present for all ages, including the 3-year-olds. As for the self-reports, an association was only found between the 15-year-olds self-report of cognitive problems and their results on the Wechsler scales.

**Conclusion:**

To identify children with cognitive problems as early as at the age of 3 years, parent-rated Pediatrics Quality of Life subscale: Cognitive Problems can be used as a screening tool. For 15-year-olds, the self-report ratings can be used as a screening tool. We also suggest a cutoff score of 80 for both the 15-year olds as well as the proxy reports. If the score falls below 80 the child should be formally evaluated using standardized cognitive test.

## Introduction

A large number of studies show that children with congenital heart defects (CHD) have higher incidence of cognitive impairments and poor academic results compared to healthy controls (1, 2). Low intellectual functioning can adversely influence many aspects of an individual’s life (3). Cognitive impairments affect not only school functioning and education (4) but also emotion regulation (5) and health (4); however, cognitive impairments affect many other aspects of daily functioning and life expectancies. Because cognitive impairments are overrepresented in children and adolescents with CHD, it is important to have reliable screening tools to identify children and adolescents in need of more extensive evaluations (3). One such possible measure is the Cognitive Problems Scale from the Pediatric Quality of Life Inventory Cardiac Module. In the current study, the aim was to evaluate whether the Cognitive Problems scale could be used as a screening tool. We investigated the association between self- as well as proxy reports on the Cognitive Problems Scale for children and adolescents with CHD for four different age groups (3-, 5-, 9-, and 15-year-olds) with their actual cognitive performance on standardized cognitive test, i.e., the Wechsler Scales of Intelligence (Swedish versions). Standardized measures of cognitive functioning, such as the Wechsler Scales of Intelligence, are time-consuming and require the person administrating the test and interpreting the test results to be a psychologist; access to a reliable and swift screening tool not requiring a psychologist would help identify the children who need to undergo standardized testing.

### Intellectual Functioning in Children with CHD

Over the last 10 years, two large meta-analyses have shown that children suffering from CHD show lower intellectual functioning than healthy controls (1, 2). However, the result is not entirely consistent since some studies show no relationship between CHD and low intellectual functioning (6, 7). This inconsistency might be explained by the fact that earlier studies investigated different levels of severity of the cardiac diagnoses. Some studies have shown a negative association between the severity of the cardiac diagnosis and intellectual functioning (8, 9), and type of cardiac diagnosis is related to certain types of cognitive difficulties (10).

An earlier study by Limbers et al. (3) investigated factors affecting self- and parental proxy reports of cognitive problems in children with CHD. There was an association between the severity of diagnosis and parental socioeconomic status (SES) with the proxy reports of cognitive problems, a finding that suggests that children from families with low SES and children with severe diagnosis should be targeted for further evaluation.

### The Association between Perceived Cognitive Problems and Actual Cognitive Functioning

Few studies have investigated the association between self- and proxy reports of cognitive ability and actual intellectual functioning as measured by standardized cognitive test batteries for children with CHD. Two studies show that there exist association between self- and proxy report of executive functioning in children with CHD (11) as well as in children with systemic lupus erythematosus (12) and the executive functioning abilities of these patients. A study by Miatton et al. (13) investigated the association between parental proxy reports and estimated full scale IQ (FSIQ) for children between 6 and 12 years with CHD: the more cognitive problems the parents reported the lower the children’s FSIQ.

Our study adds to Miatton et al.’s (13) study by also investigating the children’s own reports (self-reports) as well as their parents’ reports (proxy reports). Furthermore, we include younger children (3- and 5-year-olds). Investigating self- and proxy reports for younger children has important implications for when it is valid to start using screening tools to detect children who need to undergo further evaluation. In addition, the earlier these children are identified, the earlier interventions can be implemented in (pre)school and daily lives.

## Materials and Methods

### Participants

**Figure 1:**
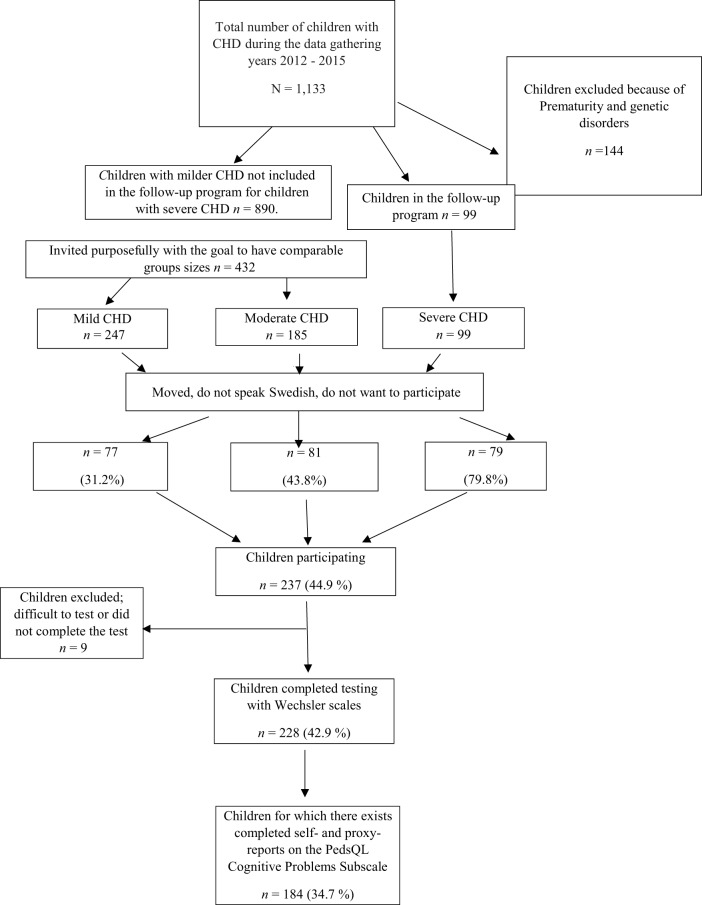
**Patients treated with surgery or by catheter interventions for CHD**.

Participants were tested over a 7-year period (2008–2015). The recruitment of the sample is illustrated in Figure 1, for more information about the recruitment of the participants, see Ref. (9). In the beginning, only children with severe CHD were recruited from the whole region of Västra Götaland. Later, children with milder CHD (a larger population) were recruited to obtain comparision groups of comparable sizes. The medical records of children living in the Västra Götaland Region (VGR) showed that 1,133 children were treated with surgery or catheter interventions for CHD at Queen Silvia Children’s Hospital in Gothenburg, Sweden during the data collection period. Of these 1,133 children, 144 children with chromosomal defects and disabilities known to influence intellectual functioning were excluded. All eligible children with severe CHD (*N* = 99) and 432 (of 890) children with milder CHD were invited. The invited families were required to speak, read, and write Swedish and to provide a signed consent. In total, 237 children and their families (44.6%) agreed to participate in the study. Participation rate was higher in the severe group than in the milder groups. All children met with a clinical psychologist at their local hospital for a psychological evaluation. Of these 237 children and their families, 228 completed testing with Wechsler Scales. Of these 228 children, self-reports and proxy ratings on the Cognitive Problems Subscale were available for 184 children.

Therefore, the target population of the current study consists of 184 children with CHD and their parents (for more demographic information about the sample, please, see Table 1). The children that were tested belonged to four different ages: 3-year-olds (*n* = 56); 5-year-olds (*n* = 34); 9-year-olds (*n* = 53); and 15-year-olds (*n* = 41). The ages for data gathering were chosen according to the follow-up program for children with severe CHD at The Queen Silvia Children’s Hospital in Gothenburg. The aim was to have a wide range of ages so the study includes cognitive testing for two preschool ages (3- and 5-year-olds) and two school ages (9- and 15-year-olds). When the youth reach the age of 18, they are transferred to the grown ups with congenital heart (GUCH) defects program. The parents of the children and adolescents had a mean age of 40.4 years (SD = 7.1) and a median income of 25,000–29,000 SEK, which indicated that the parents’ belonged to a middle class setting. Of these parents, 65% stated that they were married, 26% stated that there were co-living with a partner (not-married), and 10% stated that they were single. Approval from the ethics committee in Gothenburg, Sweden was obtained on September 20, 2011 (ref. no. 391–11).

**Table 1:** **Descriptive statistic of the participants for each age group**.

	Age group			
Variable	3-year-olds (*n* = 56)	5-year-olds (*n* = 34)	9-year-olds (*n* = 53)	15-year-olds (*n* = 41)

Mean age at testing	3 years and 1 month	5 years and 3 months	9 years and 1 month	15 years and 0 month
Gender, *n* (%)				
Male	31 (55)	11 (32)	29 (55)	17 (41)
Female	25 (45)	23 (68)	24 (45)	24 (59)
FSIQ^a^, mean (SD)	106.6 (12.9)	101.6 (13.4)	98.8 (13.3)	98.6 (14.9)
Hollingshead SES, mean (SD)	45.4 (11.5)	44.6 (11.2)	43.6 (12.6)	42.0 (13.9)
Self-reports Peds. Cog^b^ mean (SD) α	–	80.7 (25.6) 0.81	72.5 (23.4) 0.86	67.4 (24.1) 0.85
Proxy reports Peds. Cog mean (SD) α	83.0 (15.2) 0.79	82.4 (17.2) 0.87	69.8 (23.8) 0.91	67.7 (26.0) 0.93
Severity of diagnosis, *n*				
Mild	18	11	17	15
Moderate	19	14	23	16
Severe	19	9	13	10

*^a^For the 3- and 5-year-olds, WPPSI-III was used, and for the 9- and 15-year-olds, WISC-IV was used*.

*^b^The age appropriate Pediatric Quality of Life Heart module Cognitive Subscale was used*.

### Measures

#### Intellectual Cognitive Functioning

Full scale IQ was measured with Wechsler Preschool and Primary Scale of Intelligence–third edition (WPPSI-III) for the 3- and 5-year-olds (14) and with the Wechsler intelligence Scale for children–fourth edition (WISC-IV) for the 9- and 15-year-olds (15). The test is constructed to have a mean score of 100 (SD 15) in the general population and according to the normal distribution curve, 68% of children in a population should have IQ scores between 85 and 115, 28% should have IQ scores between 70 and 84 or 116 and 130, and only 4% should have extreme IQ scores, between 55 and 69 or 131 and 145.

#### Self- and Proxy Reports of Cognitive Problems

To measure perceived cognitive problems, the subscale Cognitive Problems from the PedsQL Cardiac Module 3.0 was used. There is a self-report version as well as a parental-report version of the scale. The Cognitive Problem subcale consists of only five items (the exception is the parental report version for the 2–4 year-olds which consist of 3 items). The questionnaire has been extensively validated and reliability tested internationally (16), including on a Swedish sample (17). Cronbach alpha values for child self-report in the Swedish sample was 0.50, which is surprising since we in the current study noted an alpha value above 0.80 for all three age groups as can be seen in Table 1. For the parent proxy report, the alpha value was 0.89 in the study by Sand et al. (17). In our study, the alpha value for the parent proxy reports ranged from 0.79 to 0.93 for all age groups. The self-report versions have formats appropriate for children from the age of 5 through 18. Although wording and content is highly similar between the different age groups, the tests for the different age groups are designed to be age appropriate in both language and content.

Self-reports were gathered from the 5-, 9-, or 15-year-olds, but not for 3-year-olds as they are too young for self-reports. Due to the variability of the 5- and 9-year-olds’ reading skills, the questions were read out loud by the test leader for these two age groups. The 5-year-olds indicated their answer by pointing on a scale with different “smiley faces,” and the 9-year-olds pointed out their answers using a five-point Likert scale ranging from 0 (never a problem) to 4 (almost always a problem). The 15-year-olds as well as the parents completed the form by reading it themselves and rating their answers on a five-point Likert scale ranging from 0 (never a problem) to 4 (almost always a problem). Both parents were asked to complete the form separately and were specifically told not to discuss their answers with each other. For the majority of the patients (72%), we had the rating for both parents on the PedsQL Cognitive Problems subscale so we used the mean value of these two values in our analyses. The intra-class correlation for the two parental measures was 0.86. For 22% of the patients we only had the mother’s ratings, and for 5% of the patients we only had father’s rating, and for 1% we only had ratings from one parent who had not provided gender information in the form, so for all these patients we just used this one value.

When calculating the final scores, the items are reversed and linearly transformed according to following formula provided with the PedsQL: 0 = 100, 1 = 75, 2 = 50, 3 = 25, and 4 = 0. Thus, the higher the score, the lesser the perceived cognitive problems and *vice versa*.

#### Demographic Variables

Demographic variables included the gender of the patient as well as the parents’ SES. The parents’ SES was calculated using Hollingshead Four Factor Index of Social Status (18, 19). The index uses a composite score between 3 and 66 determined by the parents’ education and occupation. For the majority of the patients (72%), we had information to calculate SES for both parents so we used the mean value of these two values in our analyses. According to the manual, when the score is present for both the parents, the mean of these two values should be used (18). For 22% of the patients we only had information about the mother’s SES, and for 5% of the patients we only had information about the father’s SES, and for 1% we had information from only one parent who had not provided gender information in the form, so for all these patients we just used this one value. We found that the parents had a mean SES of 44.0 (SD = 12.3), which is comparable with previous studies that have shown an average SES of 37.0 (SD = 11.7) in the Swedish population (20).

#### Severity of the Cardiac Diagnosis

The participants had various forms of cardiac diagnoses. These diagnoses were categorized into three diagnosis groups reflecting the severity of the diagnosis and the risk for further complications. The first group consisted of patients with *mild* severity diagnoses such as atrial septal defect, ventricular septal defect, persistent ductus arteriosus, isolated coarctation of the aorta, and pulmonary stenosis. The second group consisted of *moderate* severity diagnoses such as transposition of the great arteries, tetralogy of Fallot, complete AV-defect, total anomalous pulmonary venous drainage, and aortic stenosis. The third group consisted of *severe* diagnoses such as univentricular heart lesions, pulmonary atresia with VSD and major aortopulmonary collaterals, and patients who have undergone heart transplantation. As can be seen in Table 1, the distribution of children belonging to the three different cardiac diagnosis groups were fairly even. This does however not represent the distribution in the populations since severe cardiac diagnosis are more scarcely occurring than mild forms.

### Statistical Analysis

Statistical analyses were conducted using the IBM SPSS Statistics v. 22 software. Descriptive data of the different variables were calculated, and ANOVAs were calculated to further investigate differences between groups on the descriptive measures. For the ANOVAs, the effect size eta-square is reported: 0.01 is a small effect, 0.06 is a medium effect, and 0.14 is a large effect (21). The relevant variables all met the normality assumption, and homogeneity assumptions were also checked. When the homogeneity assumptions were not met, the Games–Howell *post hoc* test was used instead of the Bonferonni correction. Correlations were conducted for the different predictors and the outcome variable. Finally, multiple linear regression analyses were conducted to investigate the unique contribution of the different predictors in the model. In the final regression analyses, SES, severity of diagnoses, and self-ratings as well as proxy ratings were entered as predictors with FSIQ as the dependent variable. Gender was not included since no association was found between this variable and the dependent variable for any of the age groups. Hierarchical regressions were computed since they allow for evaluation of variance accounted for by the different blocks of predictors. Due to the nature of the data as well as the desire to clarify the results, hierarchical regression was calculated separately for each age group. In the first step of the hierarchical regression, the control variable SES was entered. In the next step, the dummy coded variables moderate and severe diagnoses (mild diagnosis was the reference group) were entered. In the final step, the self-reports (for the 5-, 9-, and 15-year-olds) and proxy reports were entered. The different steps in the regression were evaluated using *R*^2^ change, and the final model was evaluated by comparing the adjusted *R*^2^.

## Results

### Descriptive Statistics

Table 1 shows the means and SDs for the variables measured. All the age groups displayed an FSIQ close to 100. However, the two preschool ages had a slightly higher FSIQ. A one-way ANOVA showed that there was a significant difference in the level of FSIQ between the four age groups [*F*(3, 180) = 3.94, *p* = 0.009, η^2^ = 0.06]. This effect was of a medium size, and Bonferroni comparisons showed that the preschool children’s FSIQ was significantly higher than the school children’s FSIQ (*p* < 0.05).

The SES displayed in the four age groups is above 40, indicating that the sample on average is a middle class sample. No significant differences were found between the age groups on SES scores.

For the self-reports of cognitive problems no significant differences were found between the three age-groups that made such self-reports. For the proxy report, however, there was a significant difference between the different age groups [*F*(3, 180) = 6.85, *p* < 0.001, η^2^ = 0.10]. This effect was of a medium size, and the Games–Howell *post hoc* comparison showed that the parents of the preschool children reported significantly less cognitive problems than the parents of the school children (*p* < 0.05).

### Intra-Class Correlations between Self-Report and Proxy Report

To measure the consistency between the self- and the proxy reports, intra-class correlations were calculated using a two-way random model (ICC) for each age group (except the 3-year-olds for which no self-report exists).

For the 5-year-olds, we found no significant ICC between the self- and proxy report. For the 9-year-olds, however, we found a significant ICC of 0.74 (*p* < 0.001) between the self- and proxy report. In addition, for the 15-year-olds we found a significant ICC of 0.68 (*p* < 0.001). This result suggests that the 9- and 15-year-olds’ view of their cognitive problems are consistent with how their parents view their cognitive problems.

### Correlations between the Predictors and FSIQ

Correlations were calculated for the predictors and FSIQ for each age group (Table 2). Since severity of diagnosis was entered as an ordinal variable, where a higher value indicates a more severe diagnosis, Spearman’s rank correlation was used. Because there was no significant correlation between gender and FSIQ for any of the age groups (Table 2), this predictor was excluded from the regression analysis. As for the SES, there was a positive relation between this variable and the FSIQ for all but the 5-year-olds. For the 3-year-olds, there was a negative relation between severity of diagnosis and FSIQ, a result that indicated that the severity of diagnosis was associated with poorer intellectual functioning. No such relationship, however, was found for the older children. When it comes to the association between self-report and FSIQ, there was only a significant positive relationship for the 15-year-olds, indicating that the less perceived cognitive problems, the better their intellectual functioning. For the proxy report, we found a positive correlation for all age groups, indicating that the less cognitive problems the parent’s perceived, the higher the intellectual functioning in the children.

**Table 2:** **Spearman’s rank correlations between the predictors and the FSIQ for all age groups**.

	FSIQ			
	3-year-olds	5-year-olds	9-year-olds	15-year-olds
Gender	−0.101	−0.087	−0.172	0.145
SES	0.308*	0.238	0.338*	0.440**
Severity of diagnosis	−0.335*	−0.298	−0.183	0.075
Self-report	–	−0.156	0.266	0.456**
Proxy report	0.460**	0.368*	0.599**	0.524**

*p < 0.05.

**p < 0.01.

### Regression Analyses

To test the unique contribution and to further investigate the variance accounted for by self- and proxy reports on the dependent variable, hierarchical regression analyses were conducted separately for all age groups. SES predicted a significant amount of the variance in three of the four regression analyses (7, 13, and 20%, respectively) (Table 3). Severity of diagnosis, however, was only significantly associated with intellectual functioning for the 3-year-olds, where children with a more severe diagnosis had a significantly lower FSIQ than children with a mild diagnosis. When controlling for SES and severity of diagnosis, there still existed an association between the proxy ratings and the FSIQ for the 3-, 9-, and 15-year-olds. For the 3-year-olds, this association accounted for 13% or of the variance, and for the 9-year-olds, it accounted for 22% of the variance. For the 15-year-olds, the self- and proxy reports of cognitive problems explained 27% of the variance in the dependent variable.

**Table 3:** **Hierarchical multiple regression analyses predicting intellectual functioning for four different age groups of patients with CHD**.

	3-year-olds		5-year-olds		9-year-olds		15-year-olds	
Predictor	Δ*R*^2^	β	Δ*R*^2^	β	Δ*R*^2^	β	Δ*R*^2^	β
Step 1	0.072*		0.066		0.128**		0.201**	
SES		0.269*		0.256		0.358**		0.448**
Step 2	0.085		0.115		0.017		0.009	
Moderate diagnosis (dummy)		−0.058		−0.020		−0.134		0.073
Severe diagnosis (dummy)		−0.336*		−0.370		−0.128		−0.037
Step 3	0.131**		0.104		0.215**		0.267***	
Self-report		–		0.007		−0.152		0.170
Proxy report		0.398**		0.356		0.616***		0.443**
Total *R*^2^	0.288***		0.156		0.360***		0.477***	

*p < 0.05.

**p < 0.01.

***p < 0.001.

### Analyses Investigating a Possible Cutoff Score for the Cognitive Problem Subscale

In order to investigate which cutoff score would be appropriate when using PedsQL Cognitive Problem subscale, the children were divided into two groups. Since children achieving an FSIQ score of below 85 very often experience learning difficulties and in order to have an inclusive cutoff score criteria, children scoring below 90 in FSIQ were compared to children achieving a score of 90 and above on the FSIQ.

As can be seen in Table 4, children having a FSIQ score above 90 and thus is very unlikely of experiencing learning difficulties should have a mean value of 73.7 on the self-reports at the age of 15. For the proxy reports, these value ranges from 75.5 to 85.3 for the children with an FSIQ above 90 depending on age group. Thus, a possible inclusive cutoff score for both the 15-year-olds’ self-reports and the proxy reports for all age groups is 80. Thus, if the score falls below 80 on the Cognitive Problem subscale, the child should be formally evaluated.

**Table 4:** **Mean values and SDs for self- and proxy reports on the PedsQL Cognitive Problem Subscale for children with a FSIQ below and above 90**.

	FSIQ					
	<90			>90		
	M	SD	*n*	M	SD	*n*	*z*-Value	*p*-Value
Self-reports								
15-year-olds	48.0	20.2	10	73.7	22.0	31	2.9	0.003
Proxy reports								
3-year-olds	67.3	18.1	7	85.3	13.5	49	2.5	0.012
5-year-olds	74.0	25.3	5	83.8	15.6	29	0.8	n.s.
9-year-olds	55.4	16.4	15	75.5	24.0	38	3.1	0.002
15-year-olds	41.5	22.2	10	76.1	21.2	31	3.5	<0.001

*Mann–Whitney U tests were conducted to compare the self-reports and proxy reports of the children with an FSIQ of below 90 with those above 90*.

To further investigate what a cutoff score of 80 on PedsQL Cognitive Problems subscale would mean when used as a screening tool, odds ratios were calculated for both the self-report of the 15-year-olds and the for the proxy reports for all age groups.

The odds ratio of a 15-year-old having an FSIQ below 90 and reporting a score of below 80 on the PedsQL Cognitive Problem subscale self-report is 22.4 times more likely than the 15-year-old reporting a value above 80. Concerning the proxy reports, it is 9.4 times more likely that a child with an FSIQ below 90 would receive a proxy rating score below 80 on the Cognitive Problem subscale than above 80. Thus, the diagnostic value of using 80 can be considered fairly adequate.

## Discussion

This study aimed to evaluate whether the PedsQL Cognitive Problems subscale from the cardiac module could be used as a screening tool for identifying children with CHD who need to undergo more extensive cognitive assessments. This evaluation was done by investigating the association between children with CHD self-reports of their cognitive problems as well as their parents’ reports (proxy reports) of their children’s cognitive problems and their children’s actual FSIQ (measured using the Wechsler Intelligence Scales).

When looking at the children’s self-report, there was a strong association between the 15-year-olds’ reports of cognitive problems and their FSIQ. These results are in line with previous empirical results and metacognitive theory (22). This type of self-evaluation demands that the child has developed certain metacognitive skills. In this particular case, the children needed to have a metacognitive component referred to as *cognitive knowledge*, i.e., knowledge about themselves as learners and the factors that affect their cognition (23). The metacognitive ability of cognitive knowledge can be evident in children as young as six, but often these skills consolidate in adolescence (24). Therefore, it is not surprising that we only found an association for the 15-year-olds, since this group is the only group where the majority of the children should have fully developed this type of metacognitive ability. However, it is likely that some of the 9-year-olds had this cognitive knowledge regarding themselves although this did not affect the results on a group level. This assumption is supported by the correlation of 0.27, albeit not significant, between the 9-year-olds self-report and their FSIQ.

Regarding the proxy reports, our results showed a moderate to strong correlation between the parental rating of cognitive problems and the FSIQ for all age groups. This result agrees with the results of Miatton et al. (13), but our study adds to their results by showing that this correlation also exists for children as young as 3 and 5. This result suggests that parents have a good understanding of their very young children’s cognitive problems (i.e., as early as 3-year-olds). However, when controlling for other factors in the regression, the association between the proxy reports and the FSIQ was not significant for the 5-year-olds. This inconsistency could be a power issue due to the low number of participants (*n* = 34) in this particular age group compared to the other groups. This is evident when considering the high beta value of the proxy report predictor for the 5-year-olds compared with the other age groups.

This study lends support to the idea that both the self-report and the proxy reports of PedsQL Subscale Cognitive Problems can be used as a screening tool for identifying children who need to undergo further cognitive evaluation. Regarding the self-reports, the children need to be 15 years old for the screening tool to be valid. A suggested cutoff value for both the self- and the proxy reports is that a value below 80 on the PedsQL Cognitive Problem subscale should warrant a more formal evaluation with standardized tests.

Using the PedsQL Subscale Cognitive Problems as a screening tool is both economically sound and time-saving alternative compared to more standardized cognitive testing procedures. In addition, not all clinics have the trained staff to perform more standardized evaluations, so this type of screening tool can help these clinics identify patients who need further evaluations. Using a screening tool also enhances the possibility of testing more children from an early age. This early detection means that appropriate resources and interventions can be set in place as early as possible for children with CHD, which in turn leads to better development for the child. Cognitive abilities not only affect learning but also affect many daily functions such as emotion regulation (5) as well as health (4).

Earlier studies have shown that it is mainly children with severe cardiac diagnosis (8) who suffer from cognitive impairments, so performing extensive testing on children with milder forms of cardiac diagnosis could prove to be an insufficient and costly procedure. In these circumstances, it is highly beneficial to use the Cognitive Problems Subscale to screen children in the mild groups in order to identify those few who may experience cognitive problems and need to undergo more extensive evaluations.

A limitation of the study is that measured IQ in younger children is slightly unstable and does not necessarily reflect the intellectual functioning the child will have as an adolescent (14, 15). According to leading researchers within the field, IQ becomes stable around early adolescence (25). Although we should expect a higher variability in FSIQ in children below the age of 8, our study still found moderate association between the younger age groups and proxy reports, suggesting that a valid relationship between these measurements and the parents’ ratings of their children’s ability. Another limitation is also that the distribution of the children with different cardiac diagnosis severity was slightly biased. Although the number of children from the different diagnosis groups was fairly equal (Table 1), in real life there are more children with mild forms of CHD than children with severe cardiac diagnosis. In our study, 80% of those suffering from a severe diagnosis who were contacted about the study agreed to participate in the study. However, only 44% of the children with a moderate cardiac diagnosis and 31% with mild cardiac diagnosis agreed to participate in the study. However, because cognitive impairments are more prevalent among the children with more severe cardiac diagnoses, it is fortunate that this group is the most well represented. Although there exists a distribution bias in this study, this bias is unlikely to significantly impact the results.

In recent years, many studies have investigated the intellectual functioning of children with CHD (1, 2). This study adds to the literature by also looking at how children themselves and their parents rate their experienced cognitive problems, and how these subjective ratings are associated with the more objective measures of intellectual functioning. In addition, this study found that the screening tool PedsQL Cognitive Problems subscale was useful for identifying children and adolescents who need further cognitive interventions. Future studies should focus on trying to explain why we see an overrepresentation of children with cognitive impairments among the children with more severe diagnoses.

## Author Contributions

SB has made substantial contribution to the analysis and interpretation of the data for the work. She has been the main responsible person for the manuscript, drafted the work, and has also revised it critically for important intellectual content. CR has made substantial contributions to the conception and design of the work, the acquisition of the data, and interpretation of the work. She has also revised it critically for important intellectual content. MB has made substantial contributions to the conception and design of the work and interpretation of the work. She has also revised it critically for important intellectual content. JS has made substantial contributions to the conception and design of the work and interpretation of the work. He also made the classification of the severity of the cardiac diagnosis. He has also revised it critically for important intellectual content. All the authors (SB, CR, MB, and JS) have made a final approval of the version to be published and have agreed to be accountable for all aspects of the work in ensuring that questions related to the accuracy or integrity of any part of the work are appropriately investigated and resolved.

## Conflict of Interest Statement

The authors declare that the research was conducted in the absence of any commercial or financial relationships that could be construed as a potential conflict of interest.
